# Exosomal lncRNAs from mesenchymal stem cells as the novel modulators to cardiovascular disease

**DOI:** 10.1186/s13287-020-01812-6

**Published:** 2020-07-23

**Authors:** Ying Huang

**Affiliations:** Department of Cardiology, The First Affiliated Hospital of Anhui Medical University, Hefei, 230032 China

## Abstract

**Aim:**

Mesenchymal stem cells (MSCs) have been identified as potential therapeutic candidates for cardiovascular disease (CVD). However, the molecular mechanism underlying their therapeutic effects is unknown.

**Findings:**

Recently, Chen et al. showed that an exosomal lncRNA derived from MSCs protected against cardiomyocyte apoptosis in vivo, which provided new insight into our understanding of the functions of MSCs. Other exosomal lncRNAs from MSCs have been shown to play pivotal roles in the development of CVD.

**Conclusion:**

Exosomal lncRNAs are important molecules in the application of MSCs, which reveals, at least in part, the functional mechanism of MSCs.

Dear Editor,

Cardiovascular disease (CVD) is the leading cause of morbidity and mortality worldwide. Mesenchymal stem cells (MSCs) were identified as potential therapeutic candidates for CVD many years ago. Delivery of MSCs decreased infarcted size, improved cardiac function, regulated cardiac cell function, and provided a histological network for cell attachment. However, the underlying molecular mechanism remains to be elucidated.

Exosomes have recently been shown to be important mediators of cell-to-cell communication. Exosomes are lipid bilayer-enclosed nanovesicles containing multiple molecules, including proteins, DNAs, and RNAs, that can transfer biological information under both normal physiological and pathological conditions. Recently, Chen et al. reported that exosomal long non-coding RNA (lncRNA) derived from MSCs protected cardiomyocytes against apoptosis in vivo, which provided new insight into our understanding of the functional role of MSCs. According to the study, lncRNA-NEAT1 derived from exosomes prepared from macrophage migration inhibitory factor (MIF)-induced MSCs blocked H_2_O_2_-induced apoptosis in cardiomyocytes by modulating the expression of miR-142-3p and activating Forkhead class O1 (FOXO1). This finding suggests the potential of exosomal lncRNA-mediated therapy for clinical applications [1].

Similar to lncRNA-NEAT1, other MSC-derived exosomal lncRNAs showed activity in CVD. Exosomal metastasis-associated lung adenocarcinoma transcript 1 (MALAT1), derived from human umbilical cord MSCs, blocked aging-induced cardiac dysfunction by inhibiting the nuclear factor kappa B (NF-κB)/tumor necrosis factor-α (TNF-α) signaling pathway [2]. In addition, atorvastatin pretreatment reduced cardiomyocyte apoptosis, attenuated increases in interleukin 6 (IL-6) and TNF-α, improved cardiac function, promoted endothelial cell survival, and reduced infarct size, and these activities were associated with increased expression of the exosomal lncRNA H19 and activation of miR-675, vascular endothelial growth factor (VEGF), and intercellular adhesion molecule-1 (ICAM-1) [3]. Overexpression of the exosomal lncRNA KLF3-AS1 from MSCs decreased cell apoptosis and pyroptosis and the myocardial infarction size by suppressing miR-138-5p and regulating silent mating type information regulation 2 homolog-1 (Sirt1) expression [4].

Taken together, these findings demonstrate that exosomal lncRNAs are important molecules in the application of MSCs, and at least partially reveal the functional mechanism underlying the effects of MSCs. Nevertheless, our understanding of the precise regulatory mechanisms of exosomal lncRNAs in different cells is still in its infancy. For example, while it is known that some lncRNAs are expressed at low levels in cells but are enriched in secreted exosomes, how the exosome “captures” these lncRNA molecules is yet unknown. Although lncRNAs may have a better chance of escaping ribonuclease degradation through their association with exosomes, it is not known whether lncRNAs are modified within the exosome. In addition, lncRNAs, miRNAs, and mRNAs can be regulated by other molecules, which makes determining the regulatory relationships challenging. Therefore, further study is needed to fully understand the characteristics of exosomal lncRNAs.

## Data Availability

Not applicable.

## Abbreviations

CVD: Cardiovascular disease
MSCs: Mesenchymal stem cells
lncRNA: Long non-coding RNA
MIF: Migration inhibitory factor
FOXO1: Forkhead class O1
MALAT1: Metastasis-associated lung adenocarcinoma transcript 1
NF-κB: Nuclear factor kappa-B
TNF-α: Tumor necrosis factor-α
IL-6: Interleukin 6
VEGF: Vascular endothelial growth factor
ICAM-1: Intercellular adhesion molecule-1
MI: Myocardial infarction
Sirt1: Silent mating type information regulation 2 homolog-1
